# Interplay Between Bacterial Extracellular Vesicles and Phages: Receptors, Mechanisms, and Implications

**DOI:** 10.3390/v17091180

**Published:** 2025-08-29

**Authors:** Angelika Bołoz, Valérie Lannoy, Tomasz Olszak, Zuzanna Drulis-Kawa, Daria Augustyniak

**Affiliations:** 1 Department of Pathogen Biology and Immunology, University of Wroclaw, S. Przybyszewskiego 63/77, 50-148 Wroclaw, Poland; angelika.boloz@uwr.edu.pl (A.B.); tomasz.olszak@uwr.edu.pl (T.O.); zuzanna.drulis-kawa@uwr.edu.pl (Z.D.-K.); 2 Centre d’Immunologie et des Maladies Infectieuses (CIMI), INSERM U1135, Sorbonne Université, 75013 Paris, France; valerie.lannoy@inserm.fr

**Keywords:** bacterial extracellular vesicles (BEVs), outer membrane vesicles (OMVs), membrane vesicles (MVs), bacteriophages, phage receptors, atypical bacteria, phage-bacteria interplay

## Abstract

Bacteria and phages have coexisted for billions of years engaging in continuous evolutionary arms races that drive reciprocal adaptations and resistance mechanisms. Among the diverse antiviral strategies developed by bacteria, modification or masking phage receptors as well as their physical removal via extracellular vesicles are the first line of defense. These vesicles play a pivotal role in bacterial survival by mitigating the effects of various environmental threats, including predation by bacteriophages. The secretion of extracellular vesicles represents a highly conserved evolutionary trait observed across all domains of life. Bacterial extracellular vesicles (BEVs) are generated by a wide variety of Gram (+), Gram (−), and atypical bacteria, occurring under both natural and stress conditions, including phage infection. This review addresses the multifaceted role of BEVs in modulating bacteria–phage interactions, considering the interplay from both bacterial and phage perspectives. We focus on the dual function of BEVs as both defensive agents that inhibit phage infection and as potential facilitators that may inadvertently enhance bacterial susceptibility to phages. Furthermore, we discuss how bacteriophages can influence BEV production, affecting both the quantity and molecular composition of vesicles. Finally, we provide an overview of the ecological relevance and efficacy of BEV–phage interplay across diverse environments and microbial ecosystems.

## 1. Introduction

In both the macro and micro worlds, life on our planet depends on complex interactions and a delicate balance between organisms and their environment. To maintain this equilibrium by precise sensing of environmental conditions, cells send and receive a wide array of chemical signals, including various biomolecules. Microorganisms for this purpose utilize simple exportation membrane proteins or more sophisticated secretion systems classified into eleven types (T1SS-T11SS) [1]. Another mechanism of secretion, found both in Gram (−) and Gram (+) bacteria, involves the encapsulation of cellular components into released protein–lipid vesicles known as proteoliposomes. This unique system of secretion in Gram (−) is classified as type 0 (T0SS) [2,3]. Both Gram (−) and Gram (+) bacteria release vesicles containing components derived from their parental cells, a phenomenon also observed in extracellular vesicles (EVs) across other domains of life, including Archaea and Eukarya [4]. Similarly to EVs in higher eukaryotes, bacterial extracellular vesicles (BEVs) are believed to facilitate intercellular communication by carrying a variety of bioactive molecules, such as proteins, nucleic acids, lipids, and metabolites, contributing to communication between bacteria and their mammalian hosts [5]. Bacteria release different types of BEVs, and their secretion appears to be evolutionarily conserved, as their production has been observed across both pathogenic and non-pathogenic bacteria and in various environmental conditions [4,6]. In Gram (−) bacteria, BEVs are released through two main routes, either via outer-membrane blebbing (outer-membrane vesicles—OMVs; outer–inner-membrane vesicles—OIMVs), or alternatively, during phage infection or other stress conditions via explosive cell lysis and the random assembly of bacterial membranes (explosive outer-membrane vesicles—EOMVs; explosive outer–inner-membrane vesicles—EOIMVs). In Gram (+) bacteria, cytoplasmic membrane vesicles (CMVs) are formed both through blebbing/protrusion of the cytoplasmic membrane in a weakened peptidoglycan (PG) area, and similarly to Gram (−), they can also result from phage/stress-dependent explosive cell lysis—a pathway known as bubbling cell death, leading to the formation of explosive cytoplasmic membrane vesicles (ECMVs) [7]. Some distinctive mechanisms of vesicle release are conserved, at least among *Mycobacterium* and *Rhodococcus*—unique Gram(+) bacteria with a mycomembrane outside of the PG layer. These distinct types of vesicles are formed through blebbing of the mycolic acid-containing mycomembrane or bubbling cell death [8]. Unique BEVs are also released by atypical bacteria including members of *Mycoplasma* or *Chlamydia*, a topic that will be addressed in a later section of this article. BEV biogenesis, cargo, and function are summarized in Table 1 and extensively reviewed elsewhere [7,9,10].

BEVs facilitate the transport of unstable, insoluble, or soluble molecules in active, concentrated, and protected forms over long distances. They play a crucial role in bacterial physiology and pathogenesis, mediating both intraspecies and interspecies interactions, including those with yeasts and mammalian hosts [11,12,13]. Numerous studies have highlighted the important role of BEVs in bacterial virulence through the enhancement of microbial adherence and biofilm formation [14] or toxin delivery [15,16]. Others have documented that during the course of infection, BEVs can trigger inflammation, apoptosis, or pyroptosis in vitro and in vivo [17,18,19,20,21,22]. In line with this are numerous studies documenting that BEVs can enter host cells through various endocytic pathways, including clathrin-dependent and clathrin-independent pathways [23]. Recent research has even revealed that BEVs exploit actin-rich cellular extensions, such as filopodia and retraction fibers, to reach the host cell body prior to endocytosis [24].

Furthermore, vesicles can facilitate the temporary antibiotic resistance among pathogens either via carrying antibiotic-degrading enzymes (β-lactamases) [25] or via passive trapping of certain antibiotics, cationic peptides, or other membrane-targeting agents, thus providing cross-resistance that leads to a virulence increase [26,27,28]. There is some evidence documenting the detrimental role of BEVs in the protection of bacteria against their natural bacterial enemies—bacteriophages [29,30,31]. Finally, BEVs are involved in mediating the interaction between bacteria and their human host. Specifically, they participate in modulating the host immune response, including both the stimulation of beneficial arms of immunity [32,33] and adverse effects through immune suppression or evasion of immune system attacks [34,35,36]. Therefore, BEVs serve as a powerful bacterial weapon to evade numerous threats from the external environment, including predation by bacteriophages.

**Table 1:** Biogenesis of bacterial extracellular vesicles (BEVs) in Gram (−), Gram (+), and selected atypical bacteria.

Vesicle Type	Producing Microorganisms	Biogenesis	Composition	Known Functions	References
**OMVs** (**O**uter-**M**embrane **V**esicles)	Gram (−) bacteria (e.g., *P. aeruginosa* and *E. coli*)	Outer membrane blebbing; reduced OM-PG linkages; periplasmic pressure; LPS charge repulsion; hydrophobic insertion	Outer membrane lipids (LPS), outer membrane proteins, periplasmic proteins; typically no cytosolic content; DNA (chromosomal and plasmid) and small RNAs; metabolites	Virulence factor delivery; immune modulation; antibiotic resistance; biofilm formation; communication; innate defense blockers via sequestration of antimicrobial peptides; decoys for bacteriophages; horizontal gene transfer; metabolite delivery	[5,7,10,37]
**OIMVs** (**O**uter-**I**nner-**M**embrane **Ve**sicles)	Gram (−) bacteria (e.g., *Shewanella vesiculosa*)	Inner-membrane protrusion through weakened PG (autolysins, damage); sometimes linked to cell lysis	Outer and inner membranes, periplasmic and cytoplasmic content (DNA and RNA)	DNA transfer; horizontal gene transfer; immune interactions	[7,10]
**EOMVs** (**E**xplosive **O**uter-**M**embrane **V**esicles)	Gram (−) bacteria (e.g., *P. aeruginosa*)	Explosive cell lysis (phage-derived endolysin activation and genotoxic stress)	Outer-membrane lipids (LPS and phospholipids); outer-membrane proteins; periplasmic enzymes; occasional cytosolic hydrolases	Extracellular DNA release supporting biofilm matrix assembly and horizontal gene transfer; dispersal of cytosolic “public goods” (e.g., degradative enzymes); generation of vesicle heterogeneity in terms of sizes and composition	[38,39]
**EOIMV** (**E**xplosive **O**uter-**I**nner-**M**embrane **V**esicles)	Gram (−) bacteria (e.g., *P. aeruginosa* and *Shewanella vesiculosa* M7^T^)	Phage-encoded endolysin- or native autolysin-mediated explosive cell lysis shatters the envelope; inner- and outer-membrane fragments self-anneal into double-bilayer vesicles	Outer-membrane lipids and proteins; inner-membrane lipids and proteins; entrapped cytoplasmic cargo (genomic DNA; soluble enzymes including phage endolysins and autolysins)	Extracellular DNA release for biofilm matrix assembly and horizontal gene transfer; disperse cytosolic “public goods” (e.g., degradative enzymes); generation of vesicle heterogeneity (single- vs. double-bilayer structures)	[38,39]
**CMVs** (**C**ytoplasmic **M**embrane **V**esicles)	Gram (+) bacteria (e.g., *B. subtilis*, *S. aureus*, *Streptococcus pyogenes*)	Bubbling cell death (endolysin/autolysin-mediated PG perforation) or blebbing (e.g., via phenol-soluble modulins)	Cytoplasmic membrane, cytosolic proteins, DNA, RNA, secreted proteins	Virulence; immune modulation; communication; decoys for bacteriophages, biofilm formation; horizontal gene transfer	[7,9,10,40,41]
**ECMVs** (**E**xplosive **C**ytoplasmic **M**embrane **V**esicles)	Gram (+) bacteria (e.g., *B. subtilis* and *L. casei*)	Bubbling cell death with extended membrane damage; often prophage-triggered	Cytoplasmic membrane fragments, cytosolic proteins, DNA, endolysins	Similar functions to CMVs; delivery of intracellular virulence factors and toxins; modulation of host immune responses via cytosolic antigen presentation; facilitation of interbacterial communication and biofilm maturation; horizontal gene transfer via vesicle-associated nucleic acids	[7,9]
**mMVs** (**M**ycomembrane **V**esicles)	Mycolic acid-containing bacteria (e.g., *Corynebacterium* and *Mycobacterium*)	Mycomembrane blebbing during envelope stress (e.g., penicillin G and biotin depletion)	Mycomembrane lipids (mycolic acids) and surface proteins	Iron acquisition via vesicle-associated siderophores (only in *Mycobacterium)*	[8,42]
**IMVs** (**I**nner-**M**embrane **V**esicles)	Mycolic acid-containing bacteria (e.g., *Mycobacterium tuberculosis*)	Budding from the live cell’s cytoplasmic membrane	Inner-membrane lipids (phosphatidylinositol, phosphatidylethanolamine, cardiolipin), lipoglycan LAM, major lipoproteins (LpqH, LprG), and IM proteins	Delivery of antigenic lipoproteins and LAM to host cells; potently modulating innate immune responses	[43]
***Mycoplasma*** **EVs**	*Mycoplasma* spp.	Budding from plasma membrane of living cells; stress-induced	Cytoplasmic membrane lipids, membrane proteins, DNA, RNA	Host cell penetration; proteome modulation; immune stimulation; vesiduction (horizontal gene transfer via vesicles)	[44,45,46]
***Chlamydia*** **EVs**	*Chlamydia trachomatis*, *C. psittaci*, *C. pneumoniae*	Eversion (budding) of the inclusion membrane forming inclusion membrane-derived vesicles; increased production under stress conditions (e.g., antibiotics, IFN-γ, nutrient deprivation)	Inclusion membrane lipids, outer-membrane proteins, bacterial antigens	Early release of chlamydial antigens into host cytosol and extracellular space; modulation of host innate immunity; promotion of pro-inflammatory signaling and persistent infection; upregulated vesicle shedding under intracellular stress;	[47,48,49]

Bacteriophages, viruses that infect bacteria, are the most abundant biological entities on Earth shaping microbial diversity. These obligate parasites exhibit four common life cycles: lytic, lysogenic, pseudolysogenic, and chronic infection. Each of these phage cycles involves at least five stages: adsorption, nucleic acid injection, followed by replication and the synthesis of morphogenetic and other proteins, the assembly of virions, virion release, and further transmission [50]. The interactions between phages and their bacterial hosts are extremely complex and strongly linked to the ecological relationships of specific bacterial and viral species. Undoubtedly, phages have a major impact on the phenomenon of membrane vesicle production in bacteria, but this impact is highly variable and must be considered in the context of benefits to specific phage–host systems. This is primarily due to differences in phage replication cycles, the structure of the bacterial cell wall, the environment conditions, and the bacterial lifestyle [51].

In this review, we summarize current data on the complex role of BEVs—referred to as MVs when released by Gram (+), OMVs in Gram (−), and also produced by selected atypical bacteria in shaping bacteria–phage interactions, considering the interplay from both bacterial and phage perspectives. This review explores the dual role of BEVs, with protective activity against phages but also the capability to sensitize to phage infection. We discuss the capacity of bacteriophages to modulate the biogenesis of BEVs, altering both their quantitative output and molecular cargo. Finally, we offer an overview of the ecological significance and functional impact of BEV–phage interactions across diverse environmental niches and microbial communities.

## 2. Phage Receptors on Bacterial Extracellular Vesicles

To initiate an infection, bacteriophages must first specifically recognize and attach to their bacterial hosts. This host specificity is largely determined by bacterial surface structures such as capsular polysaccharides (CPSs), lipopolysaccharides (LPSs), proteins, pili, flagella, teichoic acids, etc., that serve as receptors, which are recognized by specialized phage-encoded receptor binding proteins (RBPs) identified as tail fibers or tailspikes [52]. Of course, there is no single type of recognition, and a phage particle must first recognize and bind to one or more receptors on the bacterial cell. In the simplest cases, a phage may use a single receptor, such as LPS bound by the KT28 and KTN6 myoviruses [53]. Sometimes phages use a primary receptor to facilitate attachment known as reversible binding but require a secondary receptor to irreversibly bind to a host cell. This scenario exists for the *Shigella flexneri* Sf6 podovirus, which first recognizes the bacterial LPS and then binds outer-membrane protein (Omp) A or OmpC [54]. Similarly, the T5 siphovirus use LPS as the primary receptor and Fhu OMP as its secondary receptor [55,56]. Likewise, the *E. coli* T4 myovirus utilizes LPS and OmpC and additionally may adsorb to *E. coli* via two distinct modes, OmpC-dependent or OmpC-independent manner [57]. Finally, phages can use two or more receptors on the bacterial surface interchangeably to bind and initiate infection, such as phage T2 infecting *E.coli* and recognizing two different outer-membrane proteins, OmpF or Ttr [58].

**Figure 1:**
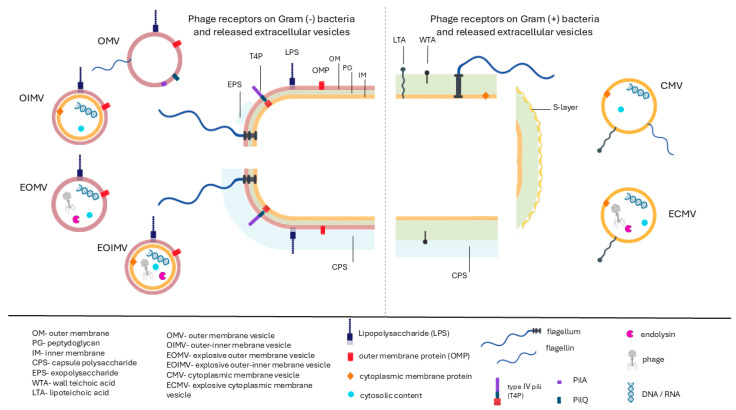
A schematic overview of bacteriophage-recognized bacterial receptors with differential distribution between the cell surface and extracellular vesicles released by the cells. In Gram (−) bacteria, confirmed cellular phage receptors include LPSs, OMPs, pili, flagellum, CPSs, and EPSs. In Gram (+), identified receptors include LTA, WTA, flagellum, proteins of the S-layer, and CPSs. Not all cellular phage receptors are preserved in bacterial extracellular vesicles (BEVs) upon release.

Due to the fundamental structural differences in the envelopes of Gram (+) and Gram (−) bacteria, as well as the presence or absence of capsules, the composition of phage receptors—both those located on the cell surface and those incorporated into BEVs released by bacteria—also differs. In Gram (−) bacteria, covered by two membranes separated by a few layers of periplasmic murein, the production of a “shield” and “cloud” composed of outer-membrane vesicles (OMVs) makes it difficult for phages to infect more cells, as the vesicles act as “bait” [30]. Since the surface of the vesicles contains the same receptors (e.g., LPSs or OMPs) as the outer cell membrane, adsorption of the phage to OMVs may (but does not have to) inactivate the phage. For Gram (+) bacteria, such a solution would be less effective, because phages specific to this group of microbes typically bind to receptors present on the surface of the thick multilayered cell wall (e.g., teichoic acids or cell wall proteins), whereas vesicles lack cell wall elements except for those directly anchored to the cytoplasmic membrane (e.g., lipoteichoic acids) [59,60]. Such vesicles could mimic the blocking function of OMVs from Gram(−) bacteria only if they retain receptors that serve as primary recognition sites for phages. However, to our knowledge, no such phenomenon has been reported to date. These general differences in phage receptor distribution are shown in Figure 1, whereas more detailed information about phages and their bacterial targets is presented in Table 2.

### 2.1. Phage Receptors in Gram (+) Bacteria and Their Membrane Vesicles (MVs)

In Gram(+) bacteria, phage receptors such as cell wall teichoic acids (WTA) [61], membrane-anchored lipoteichoic acids (LTA) [62], or flagellum [63] are typically surface-exposed molecules embedded in a thick peptidoglycan layer. Furthermore, even highly structurally conserved PG may serve as a phage receptor, for example, in *Clostridium botulinum* [64]. Interestingly, although T4P is part of a tight adherence system in Gram (+) [65], and could hypothetically be involved in phage adsorption, there is no experimental evidence on phages recognizing this particular structure, as shown in Figure 1 and Table 2. Certain phages, such as phage γ infecting *Bacillus anthracis* and phage SPP1 specific to *B. subtilis*, recognize irreversibly anchored protein receptors to the cell wall—GamR and YueB, respectively [66,67]. Highly variable and species-specific teichoic acids are the most widespread receptors targeted by phages infecting Gram (+) hosts, being notably involved in the adsorption of phages infecting *Staphylococcus* spp., *Listeria* spp., and *B. subtilis* [60]. Upon MV release, only lipoteichoic acids, which are initially anchored in membrane phospholipids, can be associated with MVs [68]. Other phage receptors exposed on the cell surface, such as capsular polysaccharides (CPSs) in *Streptococcus mutans* [69], *C. perfringens* [70], or *Tetragenococcus halophilus* [71], exopolysaccharides (EPSs) in *Streptococcus thermophilus* [72], and the S-layer protein in *C. difficile* [73], in principle, are not associated with MVs. The presence of secondary proteinaceous receptors enabling irreversible binding is also ineffective, since, without initial reversible attachment, these receptors cannot be used. Exceptions may include mycolic acid-containing bacteria, which produce MV cell envelope-associated proteins, such as proteins of the S-layer in *Mycobacteria* [8]. Summing up, vesicle formation in Gram(+) bacteria often leads to the exclusion of certain phage receptors (Figure 1, Table 2).

### 2.2. Phage Receptors in Gram (−) Bacteria and Their Outer-Membrane Vesicles (OMVs)

Conversely, because the outermost surface layer—the outer membrane of Gram (−) bacteria—closely resembles the membrane composition of the outer-membrane vesicles (OMVs) they release, these bacteria inherently incorporate phage receptor-bearing regions of the outer membrane into the forming vesicles. It was confirmed that OMVs mainly carry phage-binding sites within the LPSs in *E.coli* [29] or *P. aeruginosa* [31], and as discussed further in this paper, the interplay between OMVs and LPS-specific phages has been most extensively studied. Phage attachment is further facilitated by outer-membrane proteins (OMPs) such as OmpA and OmpC in *Shigella flexneri* [74] or OmpU in *Vibrio cholera* [30,75]. Of course, numerous other OMPs have been recognized as cellular attachment targets for phages, such as the outer-membrane transporter FhuA and the TolC efflux protein in *E.coli*, targeted by T5 and TLS bacteriophages, respectively [76,77]. Their incorporation in OMVs has also been documented [78,79]. Nevertheless, in the given examples, the OMV–phage interplay still remains to be elucidated. Thin appendages known as Type IV-pili (T4P), composed of several proteinaceous pilin subunits located in the inner membrane, periplasm, and outer membrane, may also serve as phage receptors. This can be attributed to PilA forming the pilus fiber, as well as to PilQ forming a pilus channel in the outer membrane. The T4P-based strategy of adsorption is exploited by non-lytic filamentous phages, including Pf in *P. aeruginosa* [80] or MDAΦ in *Neisseria meningitidis* [81], as well as by non-filamentous lytic phages exemplified by jumbo phages KTN4 and phiKZ in *P. aeruginosa* [82]. However, despite the presence of PilA and PilQ in *P. aeruginosa* OMVs and PilQ in *Neisseria* OMVs, as documented by a proteomic approach [83,84], their functionality in phage neutralization should be confirmed. It is worth noting that T4P systems are highly versatile, being responsible not only for adhesion but also for surface motility, microcolony formation, bacterial aggregation, biofilm production, and DNA uptake [85,86]. Therefore, their role in phage–bacteria or BEV–bacteria interactions may be broader than currently recognized. Flagella represent another group of phage receptors. As with pili, their presence in OMVs has been confirmed biochemically but not physically, for example, in the aforementioned *Salmonella enterica* serovar Typhimurium [87]. Therefore, their role as the vesicular target in phage recognition still remains unclear. Another issue involves CPSs or EPSs and their traits in shaping phage specificity. In *K. pneumoniae*, for example, CPS is recognized as the primary phage receptor by phages equipped with depolymerases, which often use LPSs, major porin, or siderophore receptors as secondary receptors to trigger infection [88]. However, these extracellularly secreted phage receptors, by definition, are absent on OMVs. Moreover, mutations that abolish capsule production (e.g., in *wbaP cps* gene cluster encoding CPSs) represent a key strategy of phage resistance in this, and probably other, encapsulated species [89,90]. Consequently, in encapsulated bacteria, OMVs appear to play a negligible role in defense against capsule-specific phages.

### 2.3. Phage Receptors in Atypical Bacteria

*Mycoplasma* spp. are among the smallest self-replicating organisms that lack a peptidoglycan-based cell wall. Instead, they possess a cholesterol-rich trilaminar membrane derived in part from the host [44]. In contrast, *Chlamydia* spp. are obligate intracellular pathogens existing in two forms: (i) infectious, non-replicative elementary bodies, (ii) metabolically active and replicative reticulate bodies. The pathogens contain a double-membrane system (outer membrane and inner membrane) and a highly reduced peptidoglycan layer, as well as many adaptations supporting their intracellular survival. One of them is replication inside the specialized membrane-bound compartments (nonacidified vacuoles) of host epithelial cells called inclusions or inclusion vacuoles [48,91].

Despite their minimalism, both genera secrete BEVs. In *Mycoplasma*, MVs originate from the cytoplasmic membrane [44], whereas in *Chlamydia*, vesicles are formed from two bacterial membranes within the inclusion environment [48]. Chlamydiaphages are members of the family *Microviridae*, with small, circular, single-stranded DNA genomes of roughly 4.5–6 kb, which bind to *Chlamydia* elementary bodies and replicate inside host inclusion vacuoles. All known chlamydiaphages (e.g., Chp1–Chp4, φCPAR39, φCPG1) share this life cycle and genome architecture [48,92]. Under β-lactam antibiotic or IFN-γ exposure, *C. trachomatis* dramatically upregulates intra-inclusion membrane vesicles, which are enriched in the major outer-membrane protein MOMP and other bacterial secreted effectors (CPAF, Pgp3, CT159, and CT166) [48]. Interestingly, it was shown that the purified major capsid protein Vp1 from the φCPG1 chlamydiaphage directly inhibited *C. trachomatis* growth in cultured epithelial cells in vitro via disrupting of host MAPK (mitogen activated protein kinase) signaling [93]. Nevertheless, the interplay of intact phage particles with chlamydial vesicles has not yet been directly observed. A significantly greater research effort is necessary to elucidate the involvement of vesicles from these atypical bacteria in their interactions with phages.

## 3. Bacterial Resistance to Phage Infection

The evolutionary arms race between bacteriophages and their bacterial hosts can take many forms, but based on a typical phage replication cycle, a few key steps can be defined that can protect bacteria from viral infection.

The most extensive research in this field is being conducted by Professor Rotem Sorek’s team, who has so far classified dozens of different phage resistance mechanisms. The main types of resistance mechanisms are grouped into five classes: (1) inhibition of phage adsorption; (2) blocking phage DNA entry into the bacterium (various superinfection exclusion systems); (3) abortive infection (signaling systems, retron systems, toxin-antitoxin systems, PrrC, bacterial gasdermins, etc.); (4) degradation of viral genetic material (restriction–modification, BREX, DISARM, CRISPRR/Cas, Argonaute systems, and many more); and (5) inhibition of phage DNA or RNA synthesis [94,95].

The primary mechanism of escaping phage infection by a sensitive bacterial population is the modification, loss, or masking of the recognized receptor. These modifications most commonly involve structural changes in bacterial surface macromolecules, such as proteins (OMPs, membrane transporters, fibriae, flagellum) and polysaccharides (LPSs, CPSs, EPSs), which are targeted by viral RBPs [96]. Since aforementioned surface structures serve as virulence agents and are recognized by the immune system as molecular recognition patterns, any modification, loss, or masking usually results in a decrease in virulence [95,97,98]. This is because phages most often recognize bacterial surface macromolecules responsible for environmental survival (e.g., LPSs, CPSs, LTA, biofilm matrix), specific adaptive processes that enable bacteria to adhere and form biofilms (e.g., fimbriae), acquire nutrients or utilize toxic substances (OMPs and other membrane transporters), and protection against the immune system or adverse physical conditions (e.g., CPSs and EPSs, biofilm matrix).

Numerous examples of the discussed bacterial defense strategy—and its consequences for bacterial fitness—have been documented. These include large deletions of LPS-related genes in *P. aeruginosa* following treatment with LPS-specific phages [99] and findings that phage-resistant strains of this species are more susceptible to antibiotics and host immune clearance mechanisms [82]. Similarly, in *K. pneumoniae*, evolutionary trade-offs in phage resistance include cross-phage sensitization and the loss of multidrug resistance [100]. Further studies are needed to clarify the relationship between bacterial fitness cost and vesicle overproduction.

**Table 2:** An overview of well-studied phages that infect non-encapsulated or encapsulated Gram (−) and Gram (+) bacteria, along with their identified receptors involved in the phage adsorption step.

Bacteria	Phage	Receptor Recognized by Phage	Reference
**Gram (−)** **non-encapsulated**	**T4** (*Escherichia virus T4*; *Tequatrovirus T4*) morphotype: myovirus	LPS (inner-core heptose and lipid A)—primary receptor for initial tethering OmpC porin—secondary receptor responsible for irreversible docking to the cell followed by DNA ejection	[57,101]
	**λ (Lambda)** (*Escherichia virus Lambda*; *Lambdavirus lambda*) morphotype: siphovirus	LamB maltoporin—essential receptor for adsorption	[102]
	**DLP1** (*Stenotrophomonas phage vB_SmaS-DLP_1*; *Septimatrevirus DLP1*) morphotype: siphovirus	Type IV pili (PilA)—essential receptor	[103]
	**χ (Chi)** (*Salmonella virus Chi*; *Chivirus chi*) morphotype: siphovirus	Flagella FLiC filament—part of receptor for reversible adsorption Flagella FLiC base—part of receptor for irreversible adsorption and DNA injection	[104]
	**Clew-1** (*Pseudomonas virus* Clew-1) morphotype: podovirus	Psl from EPS—essential receptor	[105]
**Gram (−)** **encapsulated**	**K1-5** (*Escherichia virus K1-5*; *Vectrevirus K15*) morphotype: podovirus	CPS (α-2,8-linked polysialic acid capsule K1)—primary receptor for initial anchoring LPS inner core heptose—secondary irreversible receptor that drives DNA injection	[106,107]
	**GH-K3** (*Klebsiella virus GH-K3*; *Webervirus GHK3*) morphotype: siphovirus	CPS—primary receptor for initial binding OmpC porin—secondary irreversible receptor that drives DNA injection	[108,109]
	**MDAΦ** (*Neisseria virus* MDAΦ) morphotype: filamentary	Type IV pili (PilE)—essential receptor for adsorption	[81]
	**F341** (*Campylobacter virus F341*; *Fletchervirus F341*) morphotype: myovirus	Flagella FlaA/B filament—part of receptor for reversible adsorption LOS core sugars—serve as a secondary receptor for irreversible engagement	[110]
	**PNJ1809-36** (*Escherichia virus PNJ1809-36*) morphotype: myovirus	CPS (α-2,8-linked polysialic acid capsule K1)—primary receptor for initial anchoring LPS outer core galactose—secondary irreversible receptor that drives DNA injection	[111]
**Gram (+)** **non-encapsulated**	**LL-H** (*Lactobacillus delbrueckii* subsp. *lactis virus LL-H*) morphotype: siphovirus	Lipoteichoic acid (D-alanine and α-glucosyl modifications)—essential receptor	[62]
	**Φ11** (*Staphylococcus virus Φ11*) morphotype: siphovirus	Wall teichoic acid (polyribitol-phosphate backbone with α-GlcNAc substitutions)—essential receptor for phage adsorption and DNA injection	[112,113]
	**PBS1** (*Bacillus virus PBS1*; *Takahashivirus PBS1*) morphotype: myovirus	Flagella FliC filament—essential receptor for initial step of infection	[63]
	**SPP1** (*Bacillus virus SPP1*; *Rivavirus* SPP1) morphotype: siphovirus	Wall teichoic acid (glucosylated)—primary receptor for reversible step Cytoplasmic membrane protein YueB—secondary receptor for irreversible adsorption and genome entry	[114]
	**ϕCD38-2** (*Clostridioides virus* ϕCD38-2; *Leicestervirus CD382*) morphotype: siphovirus	S-layer protein A (SlpA)—essential receptor for adsorption	[73]
**Gram (+)** **encapsulated**	**Dp-1** (*Streptococcus* virus Dp-1) morphotype: siphovirus	Wall teichoic acid enriched in choline—essential receptor for initial step of infection and DNA injection	[115]
	**A118** (*Listeria* virus A118) morphotype: siphovirus	Serovar-specific wall teichoic acid glycosylated by, e.g., N-acetylglucosamine, rhamnose—essential receptor for phage adsorption	[116]
	**CPS1** (*Clostridium virus CPS1; Gregsiragusavirus CPS1*) morphotype: podovirus	Capsular polysaccharides (CPSs)—essential receptor for adsorption	[70]

## 4. Bacterial Extracellular Vesicles—Dual Roles in Protection Against and Sensitization to Phage Infection

### 4.1. Bacterial Vesicles as Decoys During Phage Infection

Bacterial vesicles were discovered in 1965 in an auxotrophic strain of *E. coli*, under lysine-limiting growth conditions [117]. Ten years later, it was disclosed that infection by phage T4 leads to vesiculation in *E. coli* [118,119]. Whereas these mechanisms were described more than fifty years ago, reports of the role of bacterial vesicles in protection against phage predation are quite recent. In 2011, Manning and collaborators introduced the notion of extracellular vesicles in bacterial defense. According to this study, T4 phage incubated with purified OMVs drastically lost its ability to infect the susceptible *E. coli* strain ADA600, indicating that OMVs from Gram (−) bacteria irreversibly bind the phage particles, acting as decoys during phage infection [29]. Very recently, these results were confirmed in a mutant strain of *E. coli* overproducing OMVs [120].

In line with these observations, *Vibrio cholerae* OMVs are natural decoys that bind three unique virulent phages, ICP1, ICP2, and ICP3. The receptor for ICP1 and ICP3 is the LPS O1 antigen [121,122], whereas for ICP2, it is porin OmpU [75]. The phage ICP2 exhibited the lowest percentage of binding to *Vibrio* OMVs, consistent with the little expression of its receptor OmpU [30]. The authors hypothesize that this is due to the known decreased expression of OmpU by *V. cholerae* in specific in vitro conditions [123].

OMVs from *Salmonella enterica* ssp. *enterica* sv Typhimurium released by both nat-ural blebbing and as a result of mechanical cell lysis were able to bind O-antigen-specific phage P22. It turned out that phages adsorbed on both types of OMVs followed by their genome injections into the vesicle lumen, thus showing the phage-neutralizing effect of OMVs. Interestingly, naturally blebbed OMVs provide much weaker protection against phage P22 than vesicles released by mechanical lysis, which appears to result from the higher vesicle yield and greater abundance of native phage receptors in the latter [124]. Knowing that interactions between phages and vesicles depend on the presence of phage receptors in OMVs, and how important the composition of the vesicle components is for its protective value, it becomes obvious that most of the OMVs (classified according to nomenclature as EOMVs) released during phage infection are derived from the disintegrated cell envelopes formed after cell lysis. It means that the bacterial population can create a “smokescreen” to lower the number of free infective phage particles [124]. In contrast to naturally budded OMVs, some phage-induced OMVs are outer–inner-membrane vesicles (OIMVs) harboring the inner membrane of Gram (−) bacteria and some phage receptors [10]. Further analyses are required to elucidate how the OMV composition restrains phage populations.

Recently, we reported the first identification of OMVs as passive protectors of *P. aeruginosa* strain PAO1 against two LPS-specific phages, myovirus KT28 and podovirus LUZ7 (abolition of the phage lytic activity). While both phages showed effective binding to OMVs, assessed by Transmission Electron Microscopy (TEM), only the titer of myovirus KT28 was reduced in neutralization assays [31]. Our results suggest that it is not the specific receptor per se but rather the unique features of the entire phage infection mechanism that influence the OMV-dependent passive protection. Again, this work underlines the importance of understanding the first phage infection steps and mechanisms of DNA ejection, as well as OMV composition, to decipher phage neutralization.

Comparing, in general, OMV-mediated passive phage defense to other resistance strategies in terms of preserving bacterial fitness, and based on analogies with OMV-mediated resistance to peptide antibiotics (studied by Manning et al., 2021 [29]), it can be assumed that OMV production is sufficient to provide short-term protection capable of neutralizing low doses of different external stressors. Certainly, the use of membrane vesicles as a physical barrier and decoys does not impair the adaptive capacity of bacteria (no loss of valuable virulence factors). In agreement with Manning et al. [29], this may serve as a way to “buy time” until more persistent, adaptive resistance mechanisms are expressed by the bacteria.

Considering a strategy other than “passive phage neutralization”, vesicles—since they often contain DNA—may serve as horizontal gene transfer agents and spread phage-resistance systems (e.g., CRISPR/Cas system spacers enrich the recipient’s CRISPR array) [125]. From this perspective, membrane vesicles directly and indirectly protect both Gram(+) and Gram (−) bacteria against phage infection.

### 4.2. Bacterial Vesicles as Tools for Sensitization to Phage Infection

The packaging and transfer of phage receptors via bacterial vesicles serve multiple adaptive functions not necessarily related to phages. Hypothetically, BEVs from phage-sensitive bacteria can fuse with or be taken up by neighboring bacteria lacking phage-specific receptors, particularly those of the same or similar species, restoring phage susceptibility. MV-mediated receptor transfer has been clearly documented in *Bacillus subtilis*. It was shown that phage-resistant *B. subtilis* lacking the SPP1 receptor becomes susceptible again when co-cultured with phage-sensitive strains and that this transient re-sensitization (without genetic material transfer) is driven by MVs carrying receptor proteins (including YueB) [126]. However, as far as we know, no experimental studies beyond *B. subtilis* have shown extracellular vesicle-mediated transfer of functional phage receptors. Furthermore, a similar situation is encountered with Gram (−) bacteria. Although passive protection mediated by OMVs against a variety of Gram (−) species has been documented for *E. coli*, *V. cholerae*, and *P. aeruginosa*, as discussed in Section 4.1, there is currently no evidence supporting OMV-mediated phage resensitization in this group of bacteria. We have also disproved the hypothesis of transferring phage receptors by OMVs from phage-sensitive *P. aeruginosa* PAO1 to a resistant PAO1 Δ*wbpL* mutant lacking the LPS; thus, no resensitization was seen to LPS-dependent phages [31]. Likewise, another study on *E.coli* demonstrated that the exogenous addition of OMVs from the non-resistant strain MG1655∆*nlpI∆tolA* failed to restore phage sensitivity in the resistant mutant MG1655∆*nlpI∆tolA-R*, indicating that OMVs cannot effectively revert a phage-resistant strain to a phage-sensitive phenotype [120].

The limited evidence on BEV-dependent phage sensitization indicates that this phenomenon may be strictly specific to *B. subtilis*, or, more likely, it does not occur because the widespread receptor transfer is not evolutionarily beneficial to bacteria. Accordingly, from the bacterial point of view, acquiring phage receptors via vesicle-mediated transfer appears to reverse a hard-won resistance trait, reintroducing vulnerability to phage predation and providing no immediate survival advantage for the resistant population. This is supported by the fact that only around 1% of resistant cells undergo sensitization, therefore showing a rather limited effect on the population dynamics of these cells during infection [127]. Also, from an evolutionary standpoint, such random fusion of BEVs between different bacterial species would, hypothetically, be disastrous for bacteria, increasing the risk of harmful cargo delivery and uncontrolled genetic exchange. In line with this, it was recently shown that *E. coli* extracellular vesicles adhere to the surface of *Streptococcus pyogenes* and inhibit peptidoglycan remodeling, resulting in cell division defects, growth inhibition, and a decrease in pathogenicity, underlying the role of vesicles in interspecies competition [128].

The difficulties in sensitizing bacteria via the fusion of vesicles containing phage receptors with the bacterial surface do not preclude the involvement of phage-carrying vesicles in the dissemination of these phages in the bacterial population. Drawing analogies from research on BEV-dependent HGT confirmed in both Gram (−) and Gram (+) bacteria, as well as in interspecies and intraspecies contexts [129,130], it is reasonable to assume that it could also happen in the case of phages. Nevertheless, this successful transfer of phage genetic material to other bacteria would require at least (i) the encapsulation of the entire phage genome, and (ii) replication competence of the recipient. To date, there is no confirmed evidence of phage infection mediated by a vesicle containing a complete phage genome. Furthermore, there is only evidence that incomplete phage particles of temperate myoviruses (prophage of cellulose-producing *Komagataeibacter intermedius* IMBG180) and siphoviruses (*Lactococcus* phage proPhi1) can be enclosed within the vesicle lumen [131,132]. The above examples demonstrate that the potential of BEVs in the dissemination of phage infectivity remains unproven at this stage.

## 5. Effect of Phage Infection on the Production of Classical Bacterial Extracellular Vesicles

The vesiculation level can be modulated by many environmental conditions, such as temperature, nutrient availability, growth conditions, quorum sensing, and envelope targeting antibiotics [133]. Based on analogies with research on conventional antimicrobials, phages may also influence this phenomenon. Indeed, the production of BEVs is intensified in the presence of phages, primarily as a result of explosive cell lysis during virulent phage infection or when temperate phages enter the lytic cycle, driven by phage-encoded murein-degrading endolysins [39,40].

There are two predominant replication models in the bacteriophage world—the lytic cycle (carried out by virulent phages), resulting in bacterial cell death, and the lysogenic cycle (carried out by temperate phages), where the phage genome integrates into the host genome, becoming a prophage. Of course, in some exceptional conditions, prophages can be induced to enter the lytic cycle, resulting in the death of the bacterial cell by lysis anyway. An interesting feature of temperate phage genomes is the possible presence of genes advantageous to bacteria, encoding superinfection exclusion mechanisms protecting against closely related phages, as well as various virulence factor genes, serving as a so-called “gift” for the bacterial host. The benefits of lysogenic conversion (the above strategy) are usually much greater than the burden of maintaining quite large viral genomes. This is because those factors increase the adaptability and allow bacteria to colonize new niches, well-known for many human pathogens such as *Corynebacterium diphtheriae*, *V. cholerae*, Shiga toxin-producing *E. coli*, *Clostridium botulinum*, *Clostridium tetani*, *Staphylococcus aureus*, and *Streptococcus pyogenes* [134]. Since infection with temperate phages is favorable for bacteria, the issue of producing BEVs ceases to be a protective element and becomes part of a viral replicating strategy.

### 5.1. Impact of Phages on the Vesicle Number

Numerous studies have investigated the various stresses that promote prophage activation in both Gram (+) and Gram (−) bacteria under the control of the SOS pathway (inducible DNA damage repair system). Among the well-characterized effectors of prophage induction are oxidative stress, UV radiation, iron depletion, antibiotic treatment, and unfavorable pH [135]. A good example is the prophage PLE2 which, following spontaneous induction or under stress conditions, replicates its DNA and expresses proteins contributing to the production of MVs by *L. casei* BL23 during cell growth. The observation that a prophage-deficient strain exhibits reduced MV production further supports a role for this prophage in MV biogenesis [136]. In addition, BEVs themselves may indirectly trigger prophage induction by acting as carriers that initiate stress response cascades in bacterial cells, delivering compounds such as stress-related proteins, quorum-sensing (QS) signaling molecules, proteolytic enzymes, antimicrobial agents, DNA and RNA particles, toxins, and other virulence factors [137,138]. The production of vesicles in Gram (+) and Gram (−) bacteria by a pathway known as explosive cell lysis requires the activity of muralytic enzymes (e.g., endolysins), which are most often of prophage origin as demonstrated in *B. subtilis*, *S. aureus*, or *P. aeruginosa*. Of course, vesicle release through explosive cell lysis can also take place without the mediation of prophages, e.g., with autolysins or after the treatment with antibiotics [39,40,139]. Considering the involvement of phages and the crucial role of endolysin in the biogenesis of BEVs, it was documented that endolysin-deficient mutants of *P. aeruginosa* are often defective in vesicle production. In this study, the endolysin of the cryptic phage Lys (PA0629), encoded in the pyocin R and F gene clusters and induced by the RecA-mediated SOS response system, appears to be essential for the process of explosive cell lysis. In addition, the process of vesicle production and explosive cell lysis was temporally and spatially linked here [39]. Notably, there are also observations that contradict these findings. Accordingly, the deletion of the lytic module, which is an operon encoding a holin, an endolysin, and two spannins, in DLP12 cryptic prophage correlates with an increased OMV production in *E. coli*, suggesting that during evolution, this operon was domesticated to regulate vesiculation [140].

Reports on the use of phages in compassionate medicine reveal that most phage therapies are administered to patients concomitantly with antibiotics [141]. Therefore, considering that bacterial vesicles serve as a resistance mechanism to lytic phages, questions remain regarding their impact on phage–bacteria combinations. A recent study demonstrated in vitro that antibiotics combined with phage EM synergize against two multidrug-resistant (MDR) strains of *P. aeruginosa*. When treated with antibiotics, these strains show lower OMV release in the presence of phage EM, which was linked to a significantly improved bacterial eradication [142]. While only a correlation, this result merits further studies to decipher the potential role of OMV–phage–antibiotic interplay.

### 5.2. Impact of Phages on Vesicle Content

A final aspect of considering the effect of phages on BEV production is the composition of the vesicles themselves. It may vary depending on the replication cycle of the phage that infects the bacterial cell. In principle, phage-induced BMVs impact the content of the vesicles. This is evidenced by the fact that the amount of DNA associated with lysogenic *S. aureus* MVs formed by phage lysis was greater than that for MVs formed by β-lactam antibiotic-induced blebbing [139]. Consistent with these results, other studies have revealed that phage-induced BEVs carry a higher amount of DNA, including prophage DNA, and are more effective at horizontal gene transfer as documented in Antarctic bacterium *Shewanella vesiculosa* M7T [38] and *Desulfovibrio alaskensis* [143]. Although the direct mechanisms responsible for the higher DNA content in vesicles produced under the influence of phages remain unknown, it can be assumed that this may be due to disturbances in peptidoglycan synthesis or the activity of phage enzymes in this area. Defects in the peptidoglycan layer contribute to an increased DNA load in vesicles, which has been recently confirmed experimentally [144]. Apart from variations in the DNA content of the vesicles, protein composition may also be affected. In line with this, research in *A. baumannii* has shown that exogenously delivered phage lysin LysP53 stimulates the production of OMVs with a higher protein yield and lower endotoxin content compared to the naturally produced OMVs [145]. A further example involves disparities in protein profiles between MVs produced by prophage-containing and prophage-deficient bacteria [136]. It is reasonable to suggest that this phenomenon could enable bacteria to better survive in the environment. It is worth adding that some regulatory genes can be expressed by latent prophages themselves [146]. Furthermore, vesicles enriched in proteins encoded by prophages (in their latent form) are used by bacteria for their own benefit [147]. From the given examples, it follows that the most noticeable differences are between vesicles released by bacteria infected with phages in the lytic cycle versus natural (non-infected) or lysogenic conditions. The lytic cycle is rapid and results in the disintegration of bacterial cells (explosive cell lysis) and, consequently, the release of large numbers of different types of vesicles with a random content of cellular debris and a variable composition of membrane components [7,148]. It can hence be assumed that vesicles originating in this way contain the full spectrum of bacterial surface macromolecules. On the other hand, vesicles secreted by natural membrane blebbing and also during the lysogenic state seem to be much more uniform in terms of size, membrane composition, and cargo content.

Interestingly, certain bacteriophages have been shown to induce the formation of intracellular cytoplasmic vesicles in their bacterial hosts, subsequently repurposing these structures as specialized compartments to support their own replication. Recently, it was discovered that nucleus-forming phages from the *Chimalliviridae* family can actively utilize these unique vesicles for their early gene expression during the viral infection cycle. Consequently, the phage genome is initially injected into a membrane-bound, phage-generated organelle, so-called early phage infection (EPI) vesicles, which are transcriptionally active (early gene expression) and likely formed from the host inner membrane. Then the genome from the EPI vesicles is efficiently delivered to the nascent phage proteinaceous nucleus, after which the second program of gene expression (late gene expression) and DNA replication commences [149]. So far, this mechanism has been documented only for *Pseudomonas jumbo* phage phiKZ [150,151], and in 2025, for *E. coli* Goslar phage [149]. It remains to be determined, however, whether this newly proposed strategy— reminiscent of that employed by certain eukaryotic viruses that exploit endosomes initially during their replication cycle—is also present in other jumbo phages.

## 6. Effect of Phage Infection on the Production of Membrane Vesicles by Atypical Bacteria

Several investigations into vesicle secretion and phage involvement have also ex-tended to atypical bacteria. In *Mycoplasma*, prophage-encoded lipoproteins embed in the plasma membrane and likely nucleate MV budding, while genome-scale analyses uncover a wealth of cryptic prophage islands poised to contribute some new vesicle functions. *Chlamydiaphages* not only adsorb to infectious elementary bodies of *Chlamydia* and replicate within inclusions, but their capsid proteins can associate with secreted vesicles, offering potential antimicrobial strategies. In *Mycoplasma fermentans*, the φMFV1 prophage integrates at duplicated TTTTTA motifs and encodes a unique coiled-coil membrane protein, Mem, whose expression is restricted to lysogenic strains [152]. In *M. arthritidis*, the temperate phage MAV1 integrates via the attP/attB recombination process and encodes the Vir lipoprotein—a signal-peptide-containing surface protein associated with superinfection immunity [153]. Meanwhile, *M. pulmonis* P1-like phages carry an ORF8 tail-fiber protein with repetitive collagen-like motifs, which are predicted to bind bacterial Vsa surface proteins. This illustrates how prophage structural genes can contribute to host membrane architecture—potentially including those of vesicles [154].

In silico screening of 82 *M. anserisalpingitidis* genomes with PHASTER, PhiSpy, and Prophage Hunter uncovered numerous prophage-like regions of 10–20 kb that encode hallmark phage genes, integrases, capsid proteins, and tail-fiber components, suggesting a hidden reservoir of phage-derived functions within the accessory genome [155]. Although most representatives of *Mollicutes* lack canonical CRISPR/Cas defense systems, *M. cynos* strain C142 carries a complete type II-A CRISPR/Cas locus. Interestingly, several of its CRISPR spacers match prophage sequences found in other *M. cynos* isolates, documenting historical phage exposures and hinting at a role for vesicle-mediated DNA transfer (“vesiduction”) in reinforcing adaptive immunity [156,157,158].

BEV formation in *Mycoplasma* and *Chlamydia* is governed by both lipid microdomain organization (particularly cholesterol- and cardiolipin-rich regions in *Mycoplasma* and the bilayered envelope of *Chlamydia*) and by stress-induced pathways such as antibiotic exposure and interferon-γ challenge. Together, these elements enable *Mycoplasma* and *Chlamydia* to deploy virulence factors, modulate host immunity, and facilitate horizontal gene transfer [44,48].

## 7. Bacterial Vesicle–Phage Interactions in Different Ecosystems

### 7.1. Fermentation Starter Ecosystem

According to recent estimates, the current size of the global market of fermented foods is in the vicinity of USD 30 billion, with increasing trends [159]. Fermentation starters are preparations used to initiate the fermentation process in the production of various foods, such as bread, cheese, and alcoholic beverages. They consist of a culture medium, such as grains, seeds, or nutrient liquids, that has been colonized by the fermentative microorganisms. The starter culture refers to the microbial consortium responsible for carrying out fermentation, often comprising mixed species and strains. Its role is to control the communities of microbes during fermentation, standardize product quality, and reduce the risk of fermentation breakdown [160]. In bacterial fermentation, phage predation is one of the most common issues, resulting in culture failures, as well as contamination of the whole facility. A way of preventing infections is the selection of bacterial strains harboring prophages equipped with superinfection exclusion mechanisms to protect against virulent phage infection [161].

Recently, vesicles have been observed for the first time in the fermentative *Lacticaseibacillus casei* strain BL23, and most MVs were released during the first 24 h of bacterial culture. The genome of the *L. casei* BL23 strain displays six predicted prophages (PLEs), of which PLE2 circularized DNA is detected by qPCR after 24 h of standard bacterial cultures. The authors constructed a PLE2 excision-deficient *L. casei* BL23 mutant, exhibiting significantly less production of MVs than BL23 control, with or without mitomycin C-induced genotoxic stress. To conclude, prophage PLE2 plays a key role in the formation of MVs by *L. casei* BL23 [136]. Phages infecting *Lactococcus lactis* are mainly studied for their deleterious impact on industrial fermentation. However, from data obtained in the strain TIFN1 of *L. lactis*, Liu and collaborators proposed a model in which prophage proPhi1 is enveloped in MVs upon release, leaving the host intact. Very importantly, proPhi1 is a tailed siphovirus, of which enclosed particles are tailless as observed by TEM and liberated through a non-lytic process of budding. Genomic analysis of the prophage genome showed disruptions in some tail genes, explaining the incomplete phenotype [132].

### 7.2. Marine Ecosystem

In a marine ecological context, extracellular bacterial vesicles serve as an intrinsic carbon source, accumulating nutrients and supporting microbial growth. For the first time, Biller and colleagues purified vesicles from an aquatic photoautotroph, the cyanobacteria *Prochlorococcus marinus*, abundant in the aquatic environment. These vesicles supported the growth of marine heterotrophic isolates *Alteromonas* and *Halomonas*. While BEVs may not represent a large fraction of the organic carbon consumed by ocean heterotrophs, they can move fixed carbon from primary producers into the food web [162].

In addition to this crucial role, *P. marinus* BEVs also influence marine phage infection dynamics, as phage neutralization by vesicles has been observed in aquatic environments. Vesicles were mixed with a specific tailed phage PHM-2, and the phage–vesicle complexes were observed by electron microscopy. A part of the complexed phages had an altered capsid density, suggesting that they had injected their genome into the vesicles [162]. Phages are abundant in oceans, and cyanophages infecting *P. marinus* can represent a major proportion of the total marine phages [163]. Finally, the delivery of vesicles by marine cyanobacteria may decrease the probability of phage infections [162].

According to the American National Ocean Service, the frequency of coral bleaching has increased in the context of climate change. Coral bleaching is due to the rupture of symbiosis between coral and pigmented photosynthetic zooxanthellae, leading to the loss of microalgae zooxanthellae and color [164]. Likewise, *Vibrio* species are prevalent in ocean ecosystems and pose a threat to corals and other marine organisms, particularly under global warming conditions [165]. It has been discovered that bleaching of the Mediterranean coral *Oculina patagonica* results from infections by the *Vibrio shilonii* AK1 strain, detected in bleached *O. patagonica* and absent from healthy coral [164]. Furthermore, in 2016, Li et al. first isolated OMVs enriched in signaling molecules and hydrolytic enzymes from this coral-associated pathogen [166]. Very recently, it was reported that the DSM 19,607 strain of *Vibrio coralliilyticus*, another opportunistic coral pathogen, produces OMVs under in vitro conditions. When these purified OMVs were incubated with the bacterial strain and its specific myovirus SMB1 at 25 °C, they significantly reduced the phage titre in a dose-dependent manner. This suggests that in an aquatic environment, OMVs might regulate phage abundance and limit bacterial infections in coral. The authors then extracted bacteria from the mucus of *Galaxea fascicularis*, a dominant coral species in the South China Sea, and co-cultured them with MVs and viruses at 25 °C and 32 °C. No differences in bacterial abundance were observed, regardless of temperature or the presence of MVs [167]. The complex microbiome of the crystal coral *G. fascicularis* inhibits the pathogenic *V. coralliilyticus* growth [168], which could compensate for any effect in a global bacterial culture. Measuring the expansion of specific bacterial species within the culture may provide more information. This research implicates that phages and OMVs could modulate coral health and diseases, of which the molecular mechanisms must be elucidated. The authors suggest that the production of OMVs should be taken into account when phage therapies are proposed to treat coral.

### 7.3. From Orally Consumed Probiotics

The gut microbiome plays crucial physiological functions in digestion, immunity, metabolism, and maintenance of the gut barrier; therefore, dysbiosis of the gut microbiota is linked to various human diseases [169]. *Lacticaseibacillus rhamnosus*, a Gram (+) acidic bacterium belonging to the gut microbiota, is the most studied probiotic [170]. Recent studies in an in vivo model proved that MVs released by *L. rhamnosus* contribute to systemic host–microbe interactions. Male BALB/c mice were gavaged with *L. rhamnosus* JB-1 strain, and later the blood was collected for isolation of MVs that had penetrated the serum and for immunological studies [171].

Toll-Like Receptor 2 (TLR2) is an innate immune receptor distributed at the plasma membrane. When TLR2 heterodimerizes with TLR1 or TLR6, TLR2/1 or TLR2/6 complexes detect lipoteichoic acid (LTA) from membranes of Gram (+) bacteria [172]. With the cell reporter line HEK-Blue™ mTLR2, Champagne-Jorgensen et al. have determined that plasma MVs from mice fed with *L. rhamnosus* reproduce some features of the species by activating TLR2, in comparison with MVs from PBS-fed mice. The ability of plasma MVs from mice fed with *L. rhamnosus* to specifically stimulate TLR2 was confirmed with the addition of neutralizing antibodies against its LTA ligand, significantly diminishing TLR2 activation [171]. TLR2 is largely reputed to be an anti-inflammatory receptor. The protective role of TLR2 against chronic inflammation occurs through its capacity to heterodimerize with anti-inflammatory TLR10 [173]. Bone Marrow-Derived Dendritic Cells (BMDCs) preincubated with plasma MVs from mice fed with *L. rhamnosus* express more anti-inflammatory Interleukin 10 (IL-10), compared with BMDCs incubated with MVs from PBS-fed mice. This effect, abrogated by neutralizing antibodies against LTA, is TLR2-dependent. Importantly, the genomic DNA of the *L. rhamnosus* JB-1 strain contains three prophages, of which one is detected in plasma MVs from mice fed with *L. rhamnosus* [171]. These data indicate that the oral uptake of bacteria leads to the plasmatic circulation of their MVs, some of them being loaded with induced prophages, which is a newly described pathway whereby probiotics may influence the host.

## 8. Other Bacterial Vesicle–Phage Interactions

### 8.1. Bacterial Extracellular Vesicles as Biofilm Generators

According to Prof. Hans-Curt Flemming, a biofilm is “an aggregate of microorganisms, like bacteria, in which cells are frequently embedded within a self-produced matrix of extracellular polymeric substances (EPSs) and adhere to each other and/or to a surface” [174]. EPS is composed of polysaccharides, eDNA fiber-forming proteins, glycan-binding proteins, different BEVs, lipids, liquid–phage crystalline, and mineral macromolecules, all of which contribute to enhanced biofilm protection against dehydration, nutrient deprivation, and phage predation [175,176]. Among other protective mechanisms, biofilms contain “adsorption traps” like dense polymers of EPSs inhibiting the diffusion of phage particles, or dead bacteria neutralizing phage particles, as well as “metabolic refuges” in which cells in dormant phases impede phage recognition and infection or inhibit replication if already infected [177]. In addition, the anti-phage strategy is also found during the so-called “wall effect”, which occurs when phage-resistant bacteria surround sensitive ones inside the biofilm, limiting phages’ ability to lyse their target cells. Weiss et al. conducted their study in axenic mice colonized with the *E. coli* strain WG-5 and found that the oral administration of phage T7 was unable to eliminate WG-5 from the mouse gut, whereas only 20% of T7-resistant E. coli was quantified amid fecal colonies [178]. The authors explain this observation through a probable protective wall effect, which must be considered for phage therapies. On the other hand, the lysis of bacterial cells in biofilm—whether induced by phage infection, lytic enzymes, or other external stressors—leads to the accumulation of BEVs (OMVs and MVs) within the EPS. These vesicles, as cellular remnants, contribute nutrients and constitute a reservoir of DNA. Among BEVs, OMVs in particular—due to their carriage of numerous phage receptors, which are also targets of the human innate immune response—may function as decoys for both phages and the immune system. Nevertheless, lytic phages have evolved to adapt to penetrate biofilm, being equipped with virion-associated enzymes such as depolymerases, proteases, or lipases able to degrade the EPS shield (we reviewed in detail elsewhere) [176].

Prophage genes are among the most upregulated during biofilm maturation in *P. aeruginosa* [179]. In 2016, it was revealed that interstitial biofilms of *P. aeruginosa* strains PAO1 and PAK are the site of mechanical cell lysis, leading to the formation of explosive extracellular vesicles, themselves biofilm components. The team generated two mutants, PAO1Δlys and PAKΔlys, deficient for a cryptic prophage endolysin. Both are defective in OMVs within the biofilm and in biofilm development. Consequently, in planktonic *P. aeruginosa*, the prophage endolysin is involved in stress-induced vesicle expansion, as shown under anoxic growth conditions through the induction of the SOS response, though no involvement was found in aerobic cultures [39]. In conclusion, cryptic prophage endolysin-mediated mechanical lysis is an inducible mechanism for biofilm production.

### 8.2. Indirect Immunomodulatory Action of OMVs with Phages

*P. aeruginosa* infections associated with strains harboring temperate filamentous phages are linked to advanced lung disease and worse exacerbations [180]. Also, temperate filamentous phages have been associated with chronic *P. aeruginosa* wound infections. Filamentous phages typically maintain their genomes extrachromosomally and are known to establish chronic infections, characterized by the continuous release of new phage particles by the extrusion mechanism, without immediate host cell lysis. Clearly, the key to the reproductive success of these phages lies in preserving the viability of the host bacterial population; therefore, strong host immunogenicity is disadvantageous for such viruses [181].

In a murine model of acute pulmonary infection, mice inoculated with *P. aeruginosa* supplemented with phage filamentous 4 (Pf4) did not develop sepsis and displayed less inflammation than mice infected with wild-type *P. aeruginosa*, suggesting that Pf4 promotes chronic infection through immune evasion [182]. Very recently, Pennetzdorfer et al. investigated this question from *P. aeruginosa* OMVs and documented that released OMVs loaded with small RNAs remain attached to replicated Pf4 after vesiculation, leading to an altered innate immune response [183]. The role of bacterial vesicles in immunoregulation is widely described. Briefly, epithelial and immune cells detect Pathogen-Associated Molecular Patterns (PAMPs) in bacterial vesicles, triggering the secretion of pro-inflammatory cytokines [184]. In contrast, the role of bacterial vesicles bound to phages in immunomodulation remains unclear.

Recent findings demonstrate that Pf4-decorated OMVs preferentially enter endosomes following uptake by mouse and human macrophages, in comparison with OMVs alone, which would rather diffuse through the cytosol. Once within the endosomal compartment, small RNAs in Pf4-attached OMVs target the endosome-localized Toll-Like Receptor 3 (TLR3), specialized in double-stranded RNA recognition. TLR3 activation triggers the antiviral type I interferon (IFN I) liberation by human U937 macrophages, antagonizing the expression of pro-inflammatory chemokines and cytokines. Consequently, the recruitment of neutrophils by macrophages is impaired by Pf4-bound OMVs, as confirmed both in vitro and in an in vivo acute *P. aeruginosa* pneumonia mouse model [183]. These data display a new protective mechanism of bacterial vesicles, according to which Pf4-OMVs dampen inflammatory responses to endotoxins.

### 8.3. Bacterial Vesicles as a Screening Tool for Phage Specificity

In the development of phage therapy, the screening for phage specificity against bacterial strains is a challenge requiring time, as well as vast amounts of consumables and media. In some cases, the duration of laboratory procedures has to be aligned with hospital needs to treat infections by MDR clinical strains. Very recently, Bali and collaborators used the properties of OMVs as specific decoys for phages to design an in vitro screening diagnostic tool [185]. In this device, OMVs are employed to make Supported Lipid Bilayers (SLBs), the membrane-mimicking platforms formed on solid surfaces [186] that have the same natural components of bacterial outer membranes. This technology can also help researchers to identify pore-forming agents that target the outer membrane, such as defensins—antimicrobial peptides that are part of the innate immune defense—in order to develop the next generation of antimicrobials [185]. Thus, bacterial vesicles against phage infection may become an advantage by improving the workflow of phage therapies. Although the idea presented by Bali et al. is intriguing and tempting, the practical preparation of bacterial vesicles that accurately reflect the surface of diverse strains, including clinical isolates, remains highly challenging and seems to rather be a utopia in itself. In comparison, assessing phage specificity through spot assays on bacterial lawns is a more straightforward and efficient approach.

### 8.4. Bacterial Vesicles as a Potential Tool in Phage Therapy

While the direct engineering of bacterial extracellular vesicles (BEVs) for the enhanced delivery of phages, recombinant phage-derived antibacterial proteins (endolysins and depolymerases), or prophages remains largely underexplored, analogous liposome-based approaches highlight promising possibilities. For example, liposome-encapsulated phages show significantly improved gastric survival and extended persistence in the intestines of broiler chickens (in vivo) [187]. In parallel, liposome-encapsulated phages in mice demonstrate enhanced retention in the stomach and biodistribution to organs like the liver and spleen (in vivo) [188]. Additionally, liposome-trapped phages resist neutralization by antibodies and are able to enter macrophages, where they kill intracellular *Klebsiella pneumoniae* (in vitro/ex vivo) [189]. Another strategy involves liposomal delivery of endolysins, which effectively kill Gram-negative bacteria (in vitro) by overcoming outer-membrane barriers [190]. These analogies suggest that BEVs could potentially, in the future, be engineered to encapsulate phages and phage-derived products for protected delivery, carry endolysins to target MDR pathogens, or function as decoys via receptor-binding proteins—though in vivo validation remains essential.

## 9. Conclusions and Perspectives

The dynamic interplay between bacteria, their BEVs, and bacteriophages represents a complex and evolving component of microbial ecology and protective response. BEVs are involved in various aspects of bacterial defense against phage infection, acting as decoys that sequester phages, modifying surface receptor availability, and therefore, regulating the vulnerability of the bacterial population to phage attack. At the same time, emerging evidence indicates that phages can affect different routes of vesicle biogenesis, at both quantitative and qualitative levels, suggesting a bidirectional regulatory axis. While specific growth conditions may favor certain mechanisms of vesicle biogenesis, the resulting BEVs are generally heterogeneous, comprising BEVs derived from both non-lytic and explosive cell lysis-dependent routes. Their relative proportion may thus affect the outcome, with possible implications for health, ecology, and evolution.

Despite growing interest in this field, the functional implications of BEV–phage interactions in natural environments—ranging from human-associated microbiomes to aquatic ecosystems, as well as during pathogenic conditions, and involving not only typical Gram (+) and Gram (−) but also atypical microorganisms—undoubtedly require further investigations. Gaining deeper insight into the vesicle–phage interface could enhance our understanding of key processes in microbial evolution and support the development of innovative biotechnological and therapeutic approaches, such as phage therapy, microbiome modulation, and strategies to combat antimicrobial resistance.

In conclusion, BEVs are not merely passive byproducts of cell physiology or pathogenesis but active participants in microbial defense, communication, and adaptation. Their interactions with phages underscore the intricate and co-evolving relationships that shape microbial communities across ecosystems.
